# Surgical outcomes of adnexal masses classified by IOTA ultrasound simple rules

**DOI:** 10.1038/s41598-022-26441-2

**Published:** 2022-12-17

**Authors:** Erica V. Carballo, Katherine E. Maturen, Zhanhai Li, Krupa K. Patel-Lippmann, Ashish P. Wasnik, Elizabeth A. Sadowski, Lisa M. Barroilhet

**Affiliations:** 1 Department of Obstetrics and Gynecology, Vanderbilt University Medical Center, 1161 21St Avenue South, B-1126 Medical Center North, Nashville, TN 37232 USA; 2 Department of Radiology, University of Michigan, Ann Arbor, USA; 3 Department of Biostatistics and Medical Informatics, University of Wisconsin, Madison, USA; 4 Department of Radiology, Vanderbilt University Medical Center, Nashville, USA; 5 Department of Radiology, University of Wisconsin, Madison, USA; 6 Department of Obstetrics and Gynecology, University of Wisconsin, Madison, USA

**Keywords:** Anatomy, Surgical oncology, Cancer imaging, Gynaecological cancer

## Abstract

IOTA (International Ovarian Tumor Analysis) Simple Rules classifies adnexal masses as benign, malignant, or indeterminate based on sonographic features. We seek to determine if IOTA inappropriately directed women to surgery, or more aggressive surgery, than their final diagnosis warranted. This is a retrospective study of sonographically detected adnexal masses with known clinical outcomes from two institutions (n = 528). Surgically managed patients (n = 172) were categorized based on pathology and compared using Chi-square and t-test for categorical and continuous variables respectively. A logistic regression was used to predict characteristics that predicted surgery or imaging follow up of indeterminate masses. Of the 528 masses imaged, 29% (n = 155) underwent surgery for benign pathology. Only 1.9% (n = 10) underwent surgery after classification as malignant by IOTA for what was ultimately a benign mass. Surgical complications occurred in 10 cases (5.8%), all benign. Fifteen (3.2%) patients went into surgically induced menopause for benign masses, one of which was inaccurately classified by IOTA as malignant. Of the 41 IOTA indeterminate masses, the presence of soft tissue nodules on ultrasound was the only statistically significant predictor of the patient being triaged directly to surgery (OR 1.79, p = 0.04). Our findings support that the IOTA ultrasound classification system can provide clinical guidance without incurring unnecessary surgeries or surgical complications.

## Introduction

The range of pathology and clinical significance of ovarian masses varies widely from normal physiologic processes to malignancy. Pelvic ultrasound is a key component in directing patients with adnexal masses to surgical and non-surgical pathways, directing them to the appropriate surgeon and guiding the extent of surgery. For instance, in cases of malignancy there are improved outcomes and longer survival when a gynecologic oncologist performed the primary surgery^1^. Bilateral oophorectomy is indicated in many cases of malignancy, but surgically induced menopause has been implicated in an earlier decline in cognition and sexual function, and an increased risk of osteoporosis and cardiac mortality long term^2^, making preoperative pathology prediction critical to operative planning.

The IOTA (International Ovarian Tumor Analysis) Simple Rules Protocol is the ultrasound imaging risk stratification system that is the most heavily validated and commonly implemented in our region^3–5^. Table 1 lists the benign and malignant features used to classify masses in this protocol. If one or more benign features are found in the absence of any malignant features, then the tumor is defined as benign. If one or more malignant features are found in the absence of any benign features, then the tumor is defined as malignant. If no features are seen or if both malignant and benign features are observed then the tumor is indeterminate^3–6^. The reported pooled sensitivity and specificity of these rules in correctly risk stratifying masses are 93% and 95% respectively^4^. However, given the low incidence of ovarian cancer, the positive predictive value remains low raising concern for over-calling masses and intervention. Furthermore, in the indeterminate category, the rate of malignancy varies from 5 to 40%^3,7–9^, creating a risk for either unnecessary surgery, or a delay in treatment if followed conservatively.

**Table 1:** IOTA simple rules assessment.

Rules for predicting benign mass	Rules for predicting malignant mass
Unilocular cyst	Irregular solid tumor
Presence of solid components where the largest solid component has a largest diameter < 0.7 cm	Presence of ascites
Presence of acoustic shadows	At least four papillary structures
Smooth multilocular tumor with the largest diameter < 10 cm	Irregular multilocular-solid tumor with the largest diameter > 10 cm
Absent Doppler signal	High Doppler signal

In this protocol, if one or more benign features are found in the absence of any malignant features then the tumor is defined as benign. If one or more malignant features are found in the absence of any benign features, then the tumor is defined as malignant. If no features are seen or if both malignant and benign features are observed then the tumor is unclassified or indeterminate^3–5,10^.

Our primary objective is to determine if misclassification of adnexal masses by ultrasound, using the IOTA simple rules, resulted in potentially unnecessary surgeries or surgical complications for benign masses. We hypothesized some women were inappropriately directed to surgery or more aggressive surgery than the final diagnosis warranted resulting in increased operative morbidity in the form of unnecessary surgery, increased complications or increased rates of surgically induced menopause. Additionally, a secondary analysis of indeterminate masses was performed to identify characteristics that directed patients to surgery when their imaging did not clearly fall into the benign vs malignant dichotomy of IOTA.

## Methods

In this Health Insurance Portability and Accountability Act-compliant, institutional review board (IRB) approved multicenter, retrospective cohort study, subjects from the University of Wisconsin Hospital and Clinics and the University of Michigan Health System who had sonographically detected adnexal masses stratified using IOTA simple rules with known clinical outcome (surgical and/or clinical follow-up) were reviewed. All relevant guidelines and regulations have been adhered to the HIPAA guidelines recommended by the Institutional Review Boards of the University of Michigan Medical School and the University of Wisconsin Heath Sciences Institutional Review Board. Transvaginal ultrasound (TVUS) examinations from January through June 2011 were reviewed consecutively. An experienced fellowship-trained abdominal radiologist blinded to clinical outcomes reviewed ultrasound images for each patient. Women aged 18 years and older with cystic or mixed solid and cystic adnexal masses were included. Subjects were excluded if there were inadequate images available for review. Physiologic findings, including cysts smaller than 1 cm in diameter and corpora lutea, were excluded. Some small cysts > 1 cm were included because they were being followed clinically. Cysts for which there was no imaging or clinical follow up were excluded from analysis. Women with surgery or pathology diagnosis, imaging evidence of cyst resolution, imaging evidence of cyst stability for at least 2 years, or clinical follow-up including documented pelvic examination at least 2 years after TVUS were included.

We analyzed the imaging and patient characteristics of those who ultimately underwent surgery with available pathology results. Surgical patients were categorized as having a physiologic cyst, benign neoplasm or malignant neoplasm based on final pathology. Follicular and hemorrhagic cysts were classified as physiologic cysts. Endometriomas, cystadenomas, fibromas and mature teratomas were classified as benign neoplasms. The category of malignant neoplasms includes high grade serous, endometrioid and clear cell subtypes. Borderline and low-grade tumors were also categorized as malignant. The three groups were compared using Chi-square test and t-test for categorical and continuous variables respectively. For patients with bilateral adnexal masses, each mass was treated independently in our data analysis since bilateral masses had different imaging characteristics, were not necessarily given the same IOTA score or found to be same entity on final pathology. When discussing clinical outcomes on a patient level such as surgical complications or surgically induced menopause, results are reported with the number of individual patients as the denominator.

Subgroup analyses of IOTA indeterminate masses and premenopausal subjects were performed. A secondary analysis of masses classified as indeterminate by IOTA was performed to look at correlation with final pathology as well as imaging and clinical characteristics associated with direct disposition to surgery over conservative management. IOTA indeterminate masses were compared using Chi-square and t-test to identify imaging and clinical characteristics associated with direct disposition to surgery over conservative management. We used logistic regression to predict imaging follow up among IOTA indeterminate masses based on the following variables: age, menstrual status, greatest mass diameter, septum number, soft tissue nodule diameter, number of soft tissue nodules, and CA125. These features are all known predictors of malignancy. Among premenopausal women, analysis of those who underwent surgically induced menopause for benign pathology was performed. Surgically induced menopause was defined as a bilateral oophorectomy in a premenopausal patient. If LMP or definitive menopausal status was not documented in the medical record, women 50 years or younger were presumed to be premenopausal. Assuming we could expect people to undergo surgery for benign masses 20% of the time in the general population, we had a statistical power of 80% to detect a 5% increased incidence surgery performed on benign masses due to misclassification by IOTA with a sample size of 528 ultrasound studies at a significance level of p ≤ 0.05. Statistical Analysis System (SAS) software version 9.4 (Cary, NC) was used.

## Results

### Characteristics of surgically vs. conservatively managed masses

Of the 528 consecutive ultrasound studies of adnexal masses included, 172 ultimately went to surgery and 356 were followed with imaging and/or physical exam until resolution or stability (Table 2). All 172 surgically managed masses had pathologic confirmation of the diagnosis. Sixty patients had bilateral adnexal masses on imaging. Fifteen of the 60 individuals with bilateral adnexal masses underwent surgical removal of both masses. Thus, there were 468 patients imaged in total with 172 distinct masses removed from 156 patients. Patients undergoing surgical management tended to be older (46 years, SD 15), have larger masses, greater number of septa, greater number of soft tissue nodules and greater diameter of the largest soft tissue nodule present (p < 0.001; Table 2). Presence of blood flow in solid components was not common (3.5% of masses) but had a significantly increased likelihood of surgical management (p ≤ 0.001). CA-125 was not found to be a statistically significant predictor of surgical management in this cohort with a median CA125 of 23 units/mL (range 2.6–475.6) in surgically managed masses compared to median CA125 of 13.5 units/mL (range 3.7–919) in conservatively managed masses (p = 0.061). It is important to note that information regarding CA-125 was only present for 17% of subjects as it was ordered at clinicians’ discretion. Of the 172 cases that ultimately went to surgery, 60% had some form of interval imaging follow up prior to surgical intervention (Table 2).

**Table 2:** Imaging and clinical characteristics of surgically vs. conservatively managed masses.

	Surgical management (n = 172)	Conservative management (n = 356)	p-value
**Patient age (years)**	46.0 (SD:14.9)	41.2 (SD:12.2)	< 0.001
**Menstrual status**			
Premenopausal	110 (63%)	293 (83%)	< 0.001
Postmenopausal	64 (37%)	61 (17%)	
**Greatest diameter (cm)**	5.8 (SD: 3.9)	3.4 (SD: 1.4)	< 0.001
**Septum number**	0.7 (SD: 1.6)	0.2 (SD: 0.7)	< 0.001
**Number of nodules**	0.4 (SD: 0.9)	0.1 (SD: 0.3)	< 0.001
**Diameter of largest nodule (mm)**	6.7 (SD: 13.2)	1.1 (SD: 4.6)	< 0.001
**Blood flow**			
Absent	154 (90%)	353 (99%)	< 0.001
Present	18 (10%)	3 (1%)	
**Imaging follow up**			< 0.001
Yes	104 (60%)	12 (3%)	
No	68 (40%)	344 (97%)	

Of the 528 consecutive ultrasound studies of adnexal masses included, 172 ultimately went to surgery and 354 were followed with imaging and/or physical exam until resolution or stability. Patient and imaging characteristics compared above using Chi-square and t-test for categorical and continuous variables respectively. Of note, ‘septum number’ and ‘number of nodules’ were not evenly distributed, and the median values for both surgically and conservatively managed lesions in these categories are all zero. Mean values are provided within the table to provide a non-zero comparison between groups.

### Comparison of IOTA classification to final pathology of surgically managed masses

**Figure 1:**
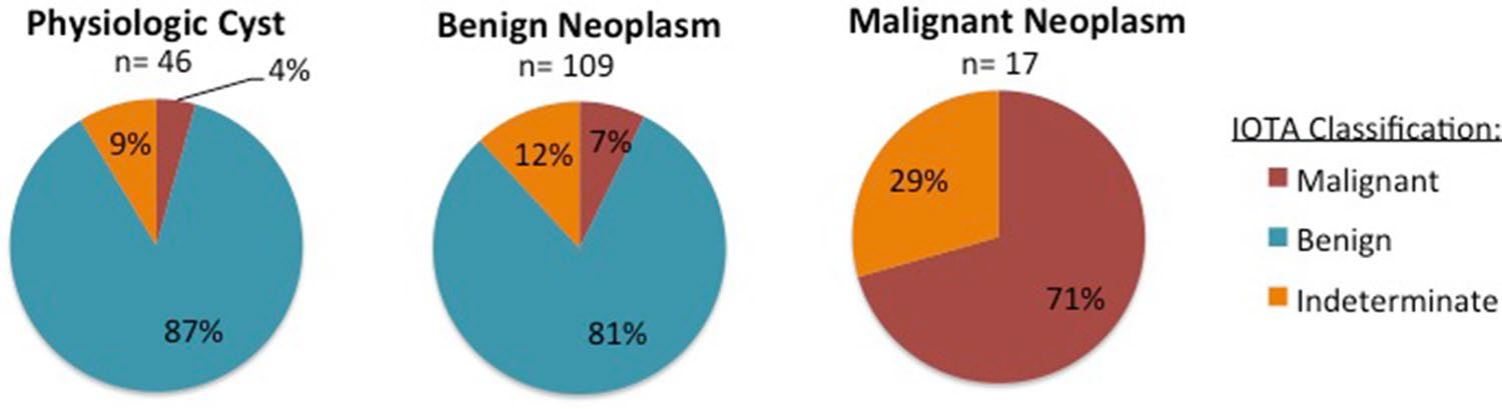
Final pathology correlates with IOTA classification: 172 adnexal masses underwent surgical removal the final pathology of which was categorized as physiologic, benign or malignant with each category represented by a chart above. 46 (27%) were physiologic cysts, 109 (63%) were benign neoplasms and 17 (10%) were malignant neoplasms. Prior to the decision to go to the OR, each of these masses was categorized by the IOTA classification system as malignant, benign or indeterminate, delineated by different colors in the charts above.

A total of 172 adnexal masses were removed from 156 subjects, the final pathology of which was categorized as physiologic, benign or malignant (Fig. 1). Forty-six (27%) were physiologic cysts, 109 (63%) were benign neoplasms and 17 (10%) malignant neoplasms (Fig. 1). None of the masses categorized as benign by IOTA classification were malignant on final pathology (p < 0.001). When looking at both physiologic cysts and benign neoplasms combined, of the 528 masses imaged, 29% (n = 155) underwent surgery for benign pathology. The majority (n = 128) were accurately classified as benign by IOTA. Of the 22 masses classified as malignant by IOTA, 12 (55%) were malignant neoplasms on final pathology, 8 (36%) were ultimately benign neoplasms (7 serous cystadenomas, 1 fibrothecoma), and 2 (9%) were physiologic cysts. Thus, there were a total of 10 masses misclassified as malignant on ultrasound that were not malignant on final pathology for a rate of 1.9% among the 528 consecutive ultrasounds reviewed, a PPV of 54.5%. The misclassification of two physiologic cysts as malignant occurred in the following circumstances: (1) The first was called malignant due to the presence of ascites in the setting of metastatic pancreatic cancer while the ovarian cyst itself was functional, and (2) the second was a follicular cyst immediately adjacent to a pedunculated fibroid that appeared to be arising from the adnexa together creating a complex appearing mass with blood flow on imaging. Of the total 17 malignant neoplasms on final pathology, 5 (29%) were indeterminate by IOTA ultrasound simple rules.

Clinical characteristics associated with malignancy included increasing age (59 ± 13 years, p ≤ 0.001), menopausal status (76% postmenopausal, p = 0.001) and elevated CA125 (mean of 143 vs. 36.6, p < 0.001). Number of soft tissue nodules (p < 0.001) and the size of the largest nodule present (p < 0.001) were both ultrasound characteristics predictive of malignancy (Table 3). Mass size and number of septa were not significant predictors of malignancy (p = 0.301 and p = 0.063 respectively). Ultrasound prediction of specific cyst type was also compared to final pathology as seen in Table 4. All masses classified as “mostly solid” (defined as > 80% solid component in the absence of any characteristic dermoid findings) were malignant.

**Table 3:** Patient and imaging characteristics of adnexal masses with histologic confirmation of diagnosis (n = 172).

	Physiologic cyst (n = 46)	Benign neoplasm (n = 109)	Malignant neoplasm (n = 17)	p-value
**Patient age (years)**	40.8 (SD:15.9)	46.2 (SD:13.6)	59 (SD: 13.0)	< 0.001
**Menstrual status**				
Premenopausal	35 (76%)	67 (63%)	4 (24%)	0.001
Postmenopausal	11 (24%)	40 (37%)	13 (76%)	
**Greatest diameter (cm)**	5.3 (SD: 3.2)	5.8 (SD: 3.8)	7.0 (SD: 5.7)	0.316
**Septum number**	0.3 (SD: 0.9)	0.9 (SD: 1.7)	0.8 (SD: 1.8)	0.077
**Number of nodules**	0.1 (SD: 0.3)	0.3 (SD: 0.8)	1.7 (SD: 1.7)	< 0.001
**Diameter of largest nodule (mm)**	3.7 (SD: 10.9)	3.7 (SD: 8.6)	25.9 (SD: 18.3)	< 0.001
**Blood flow**				
Absent	43 (96%)	103 (94%)	7 (41%)	< 0.001
Present	2 (4%)	6 (6%)	10 (59%)	
**Imaging follow up**				0.042
No	22 (48%)	65 (61%)	14 (82%)	
Yes	24 (52%)	42 (39%)	3 (18%)	

Of the patients with pathologic confirmation of their diagnosis, increasing age (p ≤ 0.001), postmenopausal status (p = 0.001) and elevated CA125 (p = 0.001) were more likely to be malignant. Mass size and number of septa were not significant predictors of malignancy (p = 0.301 and p = 0.063 respectively). Number of nodules (p < 0.001) and the size of the largest nodule present (p < 0.001) were both predictive of malignancy. Of note, ‘septum number’ and ‘number of nodules’ were not evenly distributed, and the median values across all three categories are zero. Mean values are provided within the table to provide a non-zero comparison between groups.

**Table 4:** Sonographic prediction of adnexal mass compared to final pathology.

	Physiologic cyst (n = 46)	Benign neoplasm (n = 109)	Malignant neoplasm (n = 17)	p-value
**Radiology predicted cyst type**				
Simple cyst	24 (52%)	29 (27%)	0 (0%)	< 0.0001
Hemorrhagic cyst	15 (33%)	12 (11%)	0 (0%)	0.0008
Endometrioma	0 (0%)	16 (15%)	0 (0%)	0.0036
Mature teratoma	1 (2%)	13 (12%)	1 (6%)	0.12
Complex cyst	6 (13%)	39 (36%)	12 (71%)	< 0.0001
Mostly solid (> 80%)	0 (0%)	0 (0%)	4 (24%)	< 0.0001

Masses were classified as simple cyst, hemorrhagic cyst, endometrioma, dermoid, complex cyst and mostly solid. Complex cysts were defined as masses with cystic as well as internal solid components that made up < 80% of the mass volume. Masses with > 80% solid components and no characteristic findings of dermoid were classified as “mostly solid.” All four of the masses classified as “mostly solid” on preoperative imaging were malignant. P-values were calculated by Fisher’s exact test to compare three groups.

### Analysis of surgical complications and surgically induced menopause

Surgical complications occurred in 10 cases (5.8% of patients). All surgical complications occurred in pathology confirmed benign masses. Complications included: laparoscopic cases converted to laparotomy (n = 8), pelvic hematoma (n = 1), wound seroma (n = 1) and enterotomy (n = 1). Seven (70%) were accurately classified as benign by IOTA preoperatively whereas the remaining 3 (30%) were indeterminate. One of the indeterminate masses was a hemorrhagic cyst, the surgery for which resulted in both an enterotomy and conversion to laparotomy.

Fifteen (3.2%) patients in our cohort went into surgically induced menopause for benign indications including 4 for physiologic cysts and 11 for benign neoplasms. Of premenopausal women surgically managed for benign pathology, those who underwent bilateral oophorectomy were significantly older, with an average age of 45.5 (p < 0.001). Seven (46%) of these patients had bilateral adnexal masses including 3 of the 4 individuals who underwent bilateral oophorectomy for physiologic cysts. All physiologic cysts were classified as benign by IOTA and characterized as simple cysts or classic hemorrhagic cysts on ultrasound (Table 4). Clinical factors including family history of ovarian cancer, personal history of endometriosis and patient preference were cited as reasons for bilateral oophorectomy.

Of the 11 patients who had surgically induced menopause for benign neoplasms, 4 had bilateral adnexal masses for a total of 15 masses in the 11 patients. 12 (80%) were classified as benign by IOTA, 2 (13%) were indeterminate and 1 (7%) was classified as malignant. The mass preoperatively classified as malignant was an 8 cm complex mass with a soft tissue component and blood flow in a 35-year-old woman with a CA-125 of 89 IU/L. Intraoperative frozen section was benign, but the decision was made to proceed with the planned bilateral oophorectomy due to heightened suspicion for malignancy from preoperative imaging. The mass was a benign cystadenofibroma on final pathology.

### Triage of IOTA indeterminate masses

**Figure 2:**
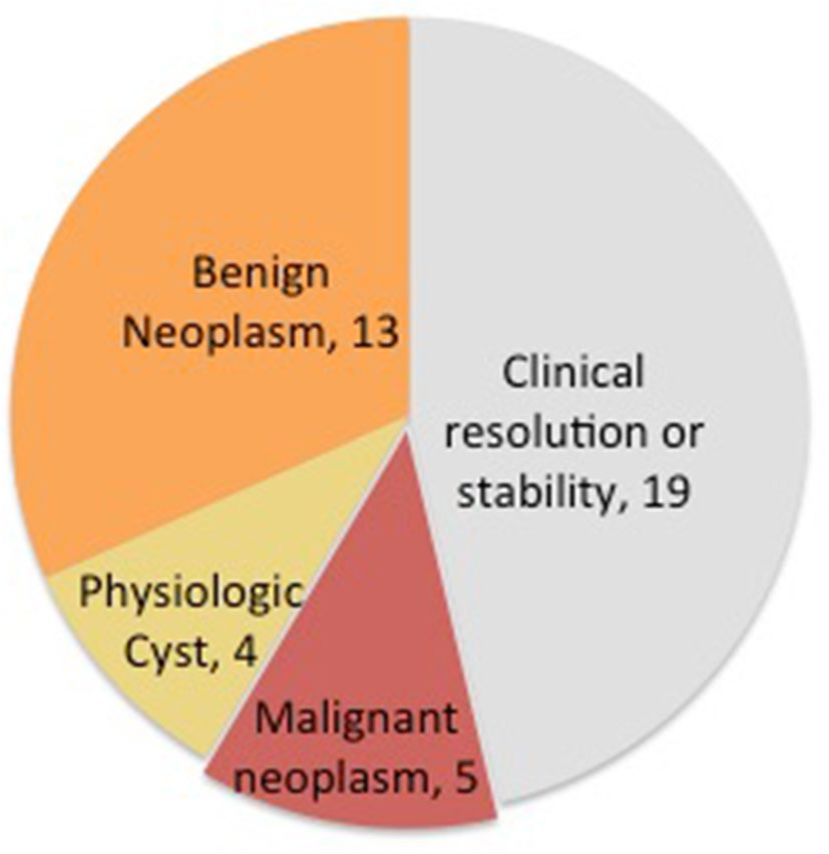
IOTA indeterminate outcomes. Of the 41 IOTA indeterminate masses 22 were eventually surgically managed. The breakdown of final pathology is shown above with the remaining 19 were followed conservatively represented in gray. 5 (12%) of indeterminate masses were ultimately malignant. Only one of these patients had follow-up imaging prior to surgical intervention due to requiring interval neoadjuvant chemotherapy.

There was not a delay in diagnosis and treatment of malignant masses classified as indeterminate by IOTA. All masses classified as indeterminate (n = 41) were complex cysts (as defined in Table 4), making up 7.8% of the images reviewed. Of these, 13 (32%) went directly to surgery while 28 (68%) had follow up imaging (Table 5). Five (12%) indeterminate masses were ultimately malignant (Fig. 2). Four of the five patients with malignant neoplasms classified as indeterminate by ultrasound were triaged directly to surgery. Surgeries took place an average of 20 days (ranging 9 to 44 days) from baseline ultrasound. The fifth patient had carcinomatosis seen on follow up CT imaging the day after her ultrasound and subsequently underwent neoadjuvant chemotherapy followed by surgical debulking.

**Table 5:** IOTA indeterminate adnexal mass characteristics.

	Surgical management (n = 22)	Conservative management (n = 19)	p-value
**Patient age (years)**	46.6 (SD: 11.8)	49 (SD: 17.7)	0.605
**Menstrual status**			0.293
Premenopausal	14 (64%)	15 (79%)	
Postmenopausal	8 (36%)	4 (21%)	
**CA125 (units/mL), median (min–max)**	23 (7.4–176)	9.5 (4.1–16)	0.0679
**Greatest diameter (cm)**	4.8 (SD: 3.3)	6.4 (SD: 3.9)	0.176
**Septum number, median (min–max)**	0 (0–5)	0 (0–2)	0.95
**Number of nodules, median (min–max)**	1 (0–5)	1 (0–2)	0.71
**Diameter of largest nodule (mm)**	14.4 (SD: 13.2)	13.1 (SD: 12.6)	0.768

Of the 41 IOTA indeterminate masses 22 were eventually surgically managed (9 had some form of follow up imaging first). The remaining 19 were followed conservatively with imaging and or exams. At 2 years of follow up, they either resolved, demonstrated satisfactory stability or were further characterized by MRI as benign. Clinical and imaging characteristics of these two groups were compared and there were no statistically significant differences.

Various patient and imaging characteristics considered by clinicians when determining disposition to surgical or conservative management were analyzed using logistic regression including age, menstrual status, CA-125, mass size, number of septa and number of nodules. The only characteristic that was associated with direct disposition to surgical management was the presence of soft tissue nodules or papillary projections on ultrasound. Each additional nodule seen increased the odds of going direct to surgery by 79% (OR [95% CI] 1.79 [1.02–3.14]; p = 0.04). Many of these masses with nodules and papillary projections did not meet IOTA criteria for malignancy due to absence of blood flow in the soft tissue component. The remaining characteristics evaluated, including age, menstrual status, mass size, presence of blood flow and CA-125 value, did not have any significant association with direct disposition to surgery. Ultimately, an additional 9 patients underwent surgery after imaging follow up for a total of 22 surgically managed IOTA indeterminate masses. At 2 years of follow up, the remaining conservatively managed masses either resolved, demonstrated satisfactory stability or were further characterized by MRI as benign.

## Discussion

We did not identify any discrete aspect of the IOTA system that led to unnecessary surgery or increased surgical morbidity in the form of operative complications, conversion to laparotomy or surgically induced menopause. Specifically, a falsely increased concern for malignancy based on ultrasound interpretation was not a major driver of surgery for benign masses or surgical complications. This is evident by our finding that of the 528 consecutive ultrasounds reviewed only 1.9% (n = 10) were misclassified as malignant by IOTA and subsequently underwent surgery for benign pathology. This is less than the 5% increase in surgery for benign masses that our study is powered to detect, making us reject our initial hypothesis. That said, these 10 masses misclassified as malignant do make up almost half of the 22 scans interpreted as malignant by IOTA criteria, creating an overall low positive predictive value (PPV = 54.5%), even in this enhanced population. No masses classified as benign were malignant on final pathology, giving this system excellent negative predictive value in our population (NPV = 100%). Our findings support consideration of non-operative management in appropriate patients with benign ultrasound morphology, consistent with the two-year interim findings of a large prospective study currently underway (IOTA5)^10^.

Increased surgical morbidity in the form of operative complications (conversion to laparotomy, pelvic hematoma, wound seroma, enterotomy) were seen in a small subset of patients. Seven (70%) of these cases were correctly risk stratified as benign preoperatively and the remaining 3 (30%) as indeterminate. All adverse events in our cohort occurred in patients having surgery for benign masses. It is important to note that this is influenced by our inclusion of conversion to laparotomy as a complication. Cases where malignancy was suspected may be more often planned open cases. Contributing factors to adverse events cited included extensive adhesive disease, impaired visualization, and excessive blood loss.

There was one case of surgically induced menopause for benign pathology where concern for malignancy based on preoperative imaging (indeterminate classification) drove the decision to remove both ovaries in a young woman with a benign cystadenofibroma. This is the only case we identified where misclassification on ultrasound led to a surgical complication or surgically induced menopause for benign pathology. All other cases of surgically induced menopause for benign masses were performed in women at increased lifetime risk for malignancy or reoperation following counseling based on accurate ultrasound risk classification. Greater weight was given to patient preference and clinical factors besides imaging in these cases. Also of note, all benign neoplasms in this category were cystadenomas or endometriomas on final pathology with 6 (40%) masses described as complex cysts on imaging, independent from IOTA classification. Mature teratomas, masses for which we are highly confident in our imaging interpretation, were universally managed with either cystectomy or unilateral oophorectomy in premenopausal women. This suggests that clinician confidence in adnexal imaging may impact preoperative counseling surrounding ovarian conservation.

For the subset of masses that were indeterminate by the IOTA classification system, presence of papillary projections was the only statistically significant predictor of malignancy and direct surgical management. Among IOTA indeterminate masses, for every increase in number of papillary projections by one, the odds of going directly to surgery increased by 79%. Many of these masses did not meet IOTA criteria for malignancy due to absence of blood flow in the soft tissue component. Even in the absence of blood flow, papillary projections are predictors of malignancy in indeterminate masses, consistent with a prior publication^7^.

This present investigation is important as clinicians caring for women with pelvic pathology decide whether IOTA, an easy to apply risk stratification system, has a role in clinical decision making as new ultrasound classification systems emerge. A critique of the IOTA system among clinicians is that it does not provide management guidance, particularly for indeterminate masses. Other studies have shown that for a subset of masses, MRI imaging following ultrasound may reduce the number of benign cysts sent for surgical evaluation^6,11,12^. The recently developed ACR’s O-RADS Ultrasound Risk Stratification and Management System provides management guidelines based on ultrasound imaging characteristics, including recommending further diagnostic imaging^13^. Early studies show similar validity between IOTA and ORADS^14,15^. IOTA remains commonly used by clinicians for its easy application and general provider familiarity, thus further study comparing these systems is warranted.

Limitations to our study include small sample size for secondary analyses, and the limitations inherent to a retrospective study approach, including the heterogeneity of ultrasound technique and advancements in ultrasound technology since the time of data collection. Strengths include a patient population encompassing two different institutions and their large catchment areas, and an experienced fellowship trained abdominal radiologist performed the ultrasound interpretations at each institution. The radiologists were blinded to the final pathology diagnosis at the time they reviewed the imaging. Furthermore, the conclusions of our study are drawn from a database of ultrasounds performed with a clinical indication and thus cannot be directly extrapolated to the use of pelvic ultrasound for ovarian cancer screening. Instances of overtreatment were reported when using pelvic ultrasound as a screening tool in the United Kingdom Collaborative Trial of Ovarian Cancer Screening (UKC-TOCS) where they found that for every one woman with detected malignancy an additional 10 women had surgery based on a false-positive^18^. No trials have been able to detect a high proportion of disease in its preclinical state or decrease mortality in long term follow up^16–18^. We do not address if applying IOTA simple rules to a cohort of screening ultrasounds would have any impact on rate of overtreatment and further study would be needed.

In conclusion, our analysis suggests that IOTA risk stratification is an effective adjunct in triaging adnexal masses, to conservative or surgical management in a way that does not increase surgical morbidity or delay treatment of malignancy. While there was not statistically significant increase in surgical morbidity of indeterminate masses with our small sample size, isolated cases were identified, making this an area where newer classification systems can provide improved clinical guidance to further improve outcomes.

## Data Availability

The datasets generated during and/or analyzed during the current study are available from the corresponding author on reasonable request.
